# Person-to-Person Cancer Transmission via Allogenic Blood Transfusion

**DOI:** 10.31557/APJCP.2021.22.3.641

**Published:** 2021-03

**Authors:** Eugen Molodysky, Ross Grant

**Affiliations:** 1 *Sydney Medical School, University of Sydney, Sydney, Australia.*; 2 *School of Medical Sciences, University of NSW, Sydney, Australia. *; 3 *Australasian Research Institute, Sydney Adventist Hospital, Wahroonga, Sydney Australia.*

**Keywords:** Circulating, tumor, blood, transfusion

## Abstract

Despite the recognized capability of Circulating Tumor Cells (CTCs) to seed tumors, allogenic blood transfusions are not presently screened for the presence of CTCs. Previous research has examined blood transfusions and the associated risk of cancer *recurrence*, but not cancer of unknown primary (CUP) *occurrence*. The Hypothesis explored in this paper proposes that there is potential for cancers to be transmitted from donor-to-patient via CTCs in either blood transfusions or organ transplants or both. This proposed haematogenic tumor transmission will be discussed in relation to two scenarios involving the introduction of donor-derived CTC’s from allogeneic blood transfusions into either known cancer surgery patients or into non-cancer patients. The source of CTCs arises either from the donor with a ‘clinically dormant cancer’ or a ‘pre-clinical cancer’ existing as yet undiagnosed, in the donor. Given the significant number of allogenic blood transfusions that occur worldwide on a yearly basis, allogenic blood transfusions have the potential to expose a substantial number of non-cancer recipients to the transmission of CTCs and associated tumor risk. This risk is greatly amplified in the low-income nations where the blood collection and processing protocols, including exclusion and screening criteria are less stringent than those in high-income countries.

## Introduction

*Cancer Survival*

The lifetime risk of a cancer diagnosis is estimated to be 1 in 2 males and 1 in 3 females in the United States. Cancer-related mortality is approximately 1 in 4 males and 1 in 5 females (National Cancer Institute, 2014). In 2004, a review of randomised clinical trials yielded disappointing results for 5-year survival benefit attributable solely to chemotherapy in adults for 22 major malignancies (National Cancer Institute, 2014). A review by Morgan et al (2004) found the overall contribution of curative and adjuvant chemotherapy to 5-year in adults to be 2.1% in the USA. A decade later, in 2014, a review of nine of the most common advanced cancers in the USA showed the median overall survival rates were short, ranging from 4-24 months, despite chemotherapy (Rajagopal et al., 2014). 

*Clinically Dormant Cancer*

Cancer dormancy refers to the prolonged clinical disease-free time between removal of the primary tumor and recurrence and is known to be a major contributor to morbidity and mortality (Lutz and Heemann, 2003; Yachida et al., 2010). 

Crowley and Seigler (1990) analysed 7,104 patients with melanoma and identified 168 who experienced their first recurrence 10 or more years after diagnosis of melanoma, for an incidence of 2.4%. The study also identified 483 patients who had 10 or more years without a subsequent recurrence. That is, of the 651 patients with a long disease-free interval, 25% (168 of 651) developed recurrent disease. Recurrent malignant melanoma has also been reported after a 35-year disease-free interval (Tahery and Moy, 1993). In breast cancer, 20% of clinically disease-free patients relapse between 17-25 years after mastectomy and, from 10-20 years, the rate being steady around 1.5% (Karrison et al., 1999; Saphner et al., 1996; Demicheli et al., 1996). 

*Pre-clinical Cancer*

Human cancers commonly progress, taking several years before a diagnosis is made, allowing a label such as ‘advanced cancer’ to mask the true pre-clinical period in which most cancers remain undiagnosed. ‘Advanced’ typically refers to patients with metastatic disease that cannot be removed or cured (Lutz and Heemann, 2003). For example, Yachinda et al., (2010) have developed a model that forecasts 3 critical times in tumour evolution. They estimate an average of 11.7 years from the initial tumorigenesis until the birth of the cell giving rise to the parental clone, an average of 6.8 years from then until the birth of the cell giving rise to the index lesion, and an average of 2.7 years from then until the patients’ death. This is consistent with the observation that 53% of pancreatic cancer patients currently present with stage IV disease, at which time it is referred to as ‘advanced’ [with metastases to distant lymph nodes or organs, and where surgical excision is not possible] (Rajagopal et al., 2014). Further, at initial diagnosis, 57% of patients initially present with ‘advanced’ or stage IV non-small cell lung cancer, while 20% of patients initially present with ‘advanced’ colorectal cancer (Rajagopal et al., 2014). Some cancers are aggressive, with significant variation. Most human cancers double their tumor volume within 60 to 700 days. The fastest growing 5% of breast cancer can reach 1-2cm within a year while the slowest growing 5% reach this size within 5 decades (Klein, 2013). On average, a 10 mm breast tumor has a median volume doubling time of 260 days, and takes 20.7 years before reaching 1 billion cells to become screen detectable (Gøtzsche et al., 2012). Conventional guidelines for the early detection of cancer recommend routine screening for individuals aged 45 and above (Smith et al., 2002), yet the pre-clinical stage may well precede this milestone, providing a prolonged period/window where early detection (and intervention) may be possible. 

*Metastasis without a primary: CUP*

Cancer of Unknown Primary (CUP) comprises a heterogeneous disease group with a diagnosis of metastatic malignancy in the absence of an identifiable primary site after a diagnostic work up, with CUPs accounting for 3-5% of all malignancies (Massard et al., 2011).

*CTCS AS SEEDS OF METASTASIS*

Paget’s 1889 conception of the ‘seed and soil’ hypothesis of cancer metastasis still holds (Fidler, 2003). Circulating Tumour cells (CTCs) were first described in1869 by an Australian, Dr Thomas Ashworth (Ashworth, 1869) and are now well documented. CTCs are blood-borne cells from either primary or secondary tumors that have migrated into the circulatory system. Over the last three decades, significant research into CTC biological properties has illuminated the critical role these cells play in the metastatic process (Young et al., 2012; O’Flaherty et al., 20102; Polzer et al., 2014). Metastasis is thought to begin with the epithelial-mesenchymal transition (EMT), a cascade of events in which tumor cells lose their ‘epithelial’ characteristics and become akin to mesenchymal cells with the ability to spread and invade tissue (Polyak and Weinberg, 2009). 

Robust evidence exists for the tumorgenicity of CTCs (Strauss and Thomas, 2010; Toloudi et al., 2011; Desai et al., 2012; Hodgkinson et al., 2014). Studies have confirmed that CTCs do in fact reflect the molecular features of cells within primary tumor masses and share the cellular and molecular features of ‘cancer-like’ stem cells (O’Flaherty et al., 2012; Polzer et al., 2009; Toloudi et al., 2011). CTCs are now accepted as the progenitors for the development of additional tumors (Toloudi et al., 2011; Gupta and Massagué, 2006), essentially constituting the ‘seeds’ for metastasis. 

With the overwhelming majority of cancer deaths, estimated at 90%, resulting from metastatic spread of the primary tumor to distant lymph nodes or organs (Christofori, 2006), newer diagnostic methods for the early diagnosis of cancer cell burden remains central to preventing metastasis and cancer-related mortality.

*CTCs as a Potential ‘Liquid Biopsy’ for Cancer*

CTC research has recently exploded as new technology platforms enable their capture, confirmation and categorisation (Nagrath et al., 2007, Andergassen et al., 2016). A growing body of evidence now confirms the presence of CTCs in the blood of cancer patients as an important indicator of the potential for metastatic disease, as well as poor prognosis (Nagrath et al., 2007; Allan and Keeney, 2010; Danila et al., 2011; Parkinson et al., 2012). Compared with the well-described difficulties of obtaining tissue biopsies from localized tumor sites, CTC as a ‘liquid biopsy’, may further provide ‘real-time’ information of metastasis in action, and hence, the patient’s disease status (Parkinson et al., 2012), thus narrowing the timeline for detecting disease progression. 

Of particular note is the emerging correlation between the number of CTCs detected and the stages of cancer, where higher CTC counts are associated with the later stages of progression (Nagrath et al., 2007; Allan and Keeney, 2010). Numerous studies have highlighted the importance of CTCs in improving clinical decision making when used in (i) early stage detection of cancer, (ii) predicting the risk of recurrence, (iii) assessing the response to treatment, (iv) selecting patients for adjuvant therapy, and (v) guiding effective treatment selection (Nagrath et al., 2007; Allan and Keeney, 2010; Danila et al., 2011; Parkinson et al., 2012; Krebs et al., 2014; Tognela et al., 2015; Ghossein et al., 1999). As blood tests are safe, cost effective and readily repeatable, information about CTC burden captured from blood samples are likely to provide a high level of clinical utility. 

*Detecting and Isolating CTCs*

Given the simplicity of routine blood sampling, testing for CTCs may be particularly useful during the pre-clinical period of cancer growth, before the overt symptoms of clinical disease emerge. While the detection of CTCs has substantial prognostic and therapeutic implications, their rarity in the circulation does impose technical challenges (Parkinson et al., 2012; Tognela et al., 2015; Ghossein et al., 1999). CTCs are estimated to be as few as 1-10 CTCs per mL of circulating whole blood (Tanaka et al., 2009), with even lower numbers in the early stages of cancer. This low frequency translates to isolating 1 cell per 109 hematologic cells in a patient with metastatic disease (Nagrath et al., 2007), rendering further analysis highly challenging. 

The challenges of detection and categorisation are further exacerbated by the difficulties associated with cancer cell heterogeneity. The phenotype of CTCs has not been fully defined (Allan and Keeney, 2010; Ghossein et al., 1999). Although CTCs were initially characterised as non-leukocytic, nucleated cells that were typically of epithelial origin, we now know that morphological features of CTCs are less well defined and may vary by cancer type, stage and treatment state (Tanaka et al., 2009; Miller et al., 2010). These challenges together coalesce to create the demand for highly sensitive, robust and standardised platforms for the capture and analysis of CTCs. In spite of these challenges, current technologies do provide reliable, though costly and often time consuming, detection of CTC’s (Bankó et al., 2019). 

Despite the well-established tumorgenicity of CTCs and abundant research interest in its clinical utility, current research has not yet investigated the role of CTCs in donor-derived cancer transmission.

*TYPES OF DONOR-DERIVED CANCER TRANSMISSION*

Person-to-person transmission of cancer may occur (1) via organ donation transmission, (2) via cell implantation transmission and (3) via blood donation transmission directly. 

1. Transmission by Organ Donation (Donor-transmitted events/cancer).

Wimmer et al. were able to show that compared to the normal population, renal transplant patients had a 4.7 times higher risk of developing CUPs (Wimmer et al., 2007). 

In 2014, transplants in the USA alone totalled 29,533 (US department of Health and Human Services).

*Clinically Dormant Cancer(s)*

Reported cases of allogenic donor-derived tumor transmission date back to the 1960’s whereby macroscopically normal renal transplants from a donor who died of metastatic disease resulted in the same carcinoma-related death of both recipients, bronchial carcinoma and epidermoid carcinoma respectively (Martin et al., 1965; McIntosh et al., 1965). As recently as 2015, Yamacake et al. described the cases of 2 kidney transplant recipients who had intestinal carcinoma from the same deceased donor.

In respect of transmission of malignancy by organ donation, Strauss et al., (2010) reported that melanoma cells can remain dormant at distant sites for decades in immune-competent patients, only to reactivate after organ transplantation to an immune-suppressed recipient. That is, the transmission of melanoma by apparent disease-free organ donors. 

‘Clinical cancer dormancy’ is frequently observed in other cancers (Spiliotaki et al., 2014; Aguirre-Ghiso, 2007). Transplant-related malignancies are known to be a major contributor of morbidity and mortality in the organ-recipient population with transplant-related malignancies developing in 15-20% of graft recipients after 10 years (Crowley and Seigler, 1990). 

The Israel Penn International Transplant Registry is among the largest and most comprehensive data base for transplant-related malignancies. Buell et al., (2004) reported as early as 2004, that there were 124 cases of confirmed cancer transmissions from 296 donors with known or incidentally diagnosed malignancies recorded in the registry from 1965 to 2003. 

*Pre-clinical Cancers(s)*

The risk of having an organ donor with undetected malignancy ranges between 1.3% and 2% (Birkeland and Storm, 2002). The 2013 report of donor-derived transmission events by the Organ Procurement Transplant Network Ad Hoc Disease Transmission Advisory Committee (DTAC) showed an increase of 43% in the number of potential donor-derived transmission events (PDDTE) reported and reviewed, when compared with 2012. There were 65 donors reported with potential malignancy events of which 5 were classified as ‘proven/probable’ transmission, with 8 affected recipients and 2 deaths (Green et al., 2015).

Retrospective analysis of the United Kingdom Transplant Registry for the period 2001 to 2010 (Desai et al., 2012), identified 15 recipients who developed donor-transmitted cancer from 13 donors. None of these donors had a history of past or active cancer at the time of the transplantation. Although all donors underwent assessment to detect transmissible diseases, cross-sectional-imaging and tumor markers were not routinely requested before donation. Desai et al. also notes in this retrospective analysis, that in a number of cases where cancer developed in the recipient. (i) donor origin was neither suspected nor investigated, and (ii) it is possible that a number of recipients may have died with a transmitted cancer that was never identified.

It has been assumed that the recipient malignancy was derived (solely) from the donor organ. However, another source is possible; this perspective postulates that the donor-derived malignancies may have been the result of the transmission of CTCs in the blood contained / entrained in the allogenic donor organ vasculature or the result of transmission of CTCs during an allogenic blood transfusion or both.

*2-Transmission by Cell Implantation of Primary Tumor Cells or CTCs*

*Patient-derived xenograft (PDX) of Primary Tumor Cells*

Several recent comparative studies suggest that patient-derived tumor growth in mice maintain many of the important characteristics of the original tumor (Lum et al., 2012; Peng et al., 2013). Donor-derived xenografting of tissue tumor cells is also known to replicate the metastatic behaviour of clinical cancer (Hiroshima et al., 2015; Decaudin, 2011). 

*Patient-derived xenograft (PDX) of CTCs*

A significant recent development in cancer research has been the detection, isolation and characterisation of CTCs, with CTCs being used to generate PDX experimental models of cancer.

Blood samples from 6 patients with chemotherapy-naïve, extensive-stage small-cell lung cancer (SCLC) were enriched for CTCs and implanted into one or both flanks of immunocompromised mice, resulted in the formation of SCLC tumors (Hodgkinson et al., 2014). Critically, this study was able to demonstrate preservation of morphological and genetic characteristics. Toyoshima et al. (2015) showed that tumor-initiating cells are present in the peripheral blood of advanced gastric cancer patients through the establishment of in vivo transplantable tumors in mice from patients CTCs. CTCs have also been used to generate other cancers including breast cancer and prostate cancer PDX models (Baccelli et al., 2013; Yu et al., 2014; Vidal et al., 2015; Williams et al., 2015).

*3- Transmission by Blood Donation*

*Autologous Blood Transfusions / Intra-operative blood salvage (IBS)*

The risk of tumor transmission via blood transfusion has been the subject of past research. While the difference in the risk of cancer recurrence arising from autologous and allogenic blood transfusions has been examined, research has primarily examined the association with the risk of cancer recurrence but not CUP occurrence (Salunkhe et al., 2015; Vamvakas and Blajchman, 2009; Klein, 1995; Li et al., 2015; Waters et al., 2012; Vamvakas, 1995; Gakhar et al., 2013; Heiss et al., 1994). Numerous meta-analyses of intra-operative blood salvage (IBS) in cancer surgery exist (Li et al., 2015; Waters et al., 2012; Vamvakas, 1995; Zhai and Sun, 2013). However, these focus on primary site cancer cells shed during the surgical procedure, which are distinct from blood-borne CTCs. Of note, to date, no study has been conducted to examine the role of donor-derived CTCs in facilitating a CUP in the recipient, following an allogenic blood transfusion.

*Allogenic Blood Transfusions*

Intraoperative blood salvage (IBS) is a technique frequently employed in major blood loss surgery, however it has been restricted in cancer surgery patients because of contaminating cancer cells ‘shed’ during the surgery and their systemic dissemination following reinfusion.

 For the majority of patients requiring blood transfusion, the source is allogenic, that is, donor-derived (Salunkhe et al., 2015). It is estimated that each year 118.5 million blood donations are collected globally (World Health Organization, 2020).

Since the end of the Second World War, blood banks have inclined towards fractioning whole blood into components (red blood cell, plasma and platelets) for economic efficiency and logistical longevity (Murdock et al., 2014). Leukocyte reduction via filtration is less effective on whole blood compared to pre-separated blood components (Bruil et al., 1995; Singh and Kumar, 2009), rendering whole blood transfusion recipients at a relatively higher risk of encountering CTCs. According to the World Health Organisation, whole blood 60% of the total number of transfusions, defined as blood ‘transfused to a patient in an unmodified state’ occur in low or middle-income countries, countries (World Health Organization, 2020). This means lower to middle income countries are disproportionately placed at risk of receiving CTCs via blood that is not leuko-reduced. 

Further, whole blood transfusion is still routinely used in military circumstances and austere environments where a well-equipped blood bank is not accessible (Auten et al., 2015; Beckett et al., 2015). In addition, whole blood transfusion has been found to possess superior therapeutic benefits over blood components in paediatric cardiac surgery and shock resuscitation (Murdock et al., 2014; Jobes et al., 2015; Rhee et al., 2015), which merit its continued usage. 

The Australian Red Cross Blood Service (ARCBS) tests for five transfusion transmissible diseases (HIV/AIDS, Hepatitis B, Hepatitis C, human T-cell lympho-proliferative virus [HTLV] and syphilis). The ARCBS excludes donors with a history of cancers such as leukaemia, lymphoma and myeloma, however ARBCS does accept people who remain free of cancer for five years after the completion of their treatment. In contrast, the American Red Cross Blood Service excludes donors of cancers of the blood, but does accept donations from patients with solid cancers after 12 months have passed since successful treatment (Catling et al., 2008). Current recommendations set by WHO for safe screening of donor blood products state that, “For individuals with a past history of solid malignant tumour, BTS (blood transfusion services) may consider acceptance if 5 years or more since completion of successful curative treatment (WHO, 2012).

This Perspective postulates that the current practices for allogenic blood transfusions may place recipients at risk of tumor transmission. This stems primarily from two factors:

Firstly, the current blood donor selection criteria includes, not excludes, individuals who remain disease-free of cancer for one years after the completion of their cancer treatment in the USA and 5 years in Australia and those countries adopting the WHO recommendations (Australian Red Cross Blood Service; Catling et al., 2008, WHO 2012). As ‘clinical dormancy’ represents a reservoir from 10 to 30 years (Strauss and Thomas, 2010; Spiliotaki et al., 2014), the current selection criteria may allow relapsing cancer patients to donate blood. Further the failure to diagnose cancer in its pre-clinical phase, especially as evidenced and represented by the plethora of ‘advanced cancer’ diagnoses, may again allow for pre-clinical cancer patients to also donate blood (and organs).This situation is most likely to be compounded in the lower-income nations.

Secondly, current filtration practices for leukocyte reduction remove only 80% to 99% of leukocytes (Singh and Kumar, 2009). Previous experiments into leukocyte filtration show inconsistent results for complete removal of primary site tumor cells shed during surgery (Bruil et al., 1995; Catling et al., 2008; Edelman et al., 1996). As the use of leukocyte reduction filters are unable to guarantee complete elimination of contaminating cancer cells shed during surgery from the reinfused blood (Hansen et al., 1993; Hansen et al., 1997), it is likely that the standard leukocyte reduction filters used by blood banks are also unable to guarantee complete elimination of CTCs. In particular, previous evidence used to purport the effective removal of tumor cells in blood after leukocyte reduction, has been criticised as flawed. Criticisms range from inferior detection sensitivity, to overestimating tumor cell reduction, to mistakenly generalising tumor cell filtration in plasma as identical with filtration in blood (Hansen, 2006). 

The likelihood of incomplete CTC elimination is increased by the fact that CTCs are heterogeneous, yet not dissimilar in size to leukocytes (Allan and Keeney, 2010) with the overlap in size favouring the smaller CTCs. Whole blood donations are not currently screened for CTCs.

The risk of recipients developing cancer from donor-derived CTCs may depend on both the number and characteristics of the CTCs, as well as the vulnerability of the recipient’s immune system that places the recipient at additional risk. 

While irradiation of allogenic blood is typically reserved for the immune-compromised, its use has been demonstrated to provide the efficient elimination of shed cancer cells contaminating the blood salvaged for autologous blood transfusion, following irradiation with 50 Gy (Hansen et al., 1999). While CTCs from donors in cancer ‘remission’ or from donors with pre-clinical cancer represent a real risk for all recipients who are immune compromised a degree of risk may also extend to otherwise healthy individuals who exhibit a degree of immune incompetence such as reduced natural killer (NK) cell function. NK cells are particularly important in recognising and destroying cancer cells including CTC’s and reduced NK activity has been shown to increase the risk of tumor development (Wu and Lanier, 2003; Moon and Powis, 2019; Vidal et al., 2019). Importantly a significant reduction in NK activity has been linked to multiple lifestyle factors including, obesity (O’Shea and Hogan, 2019), psychosocial stress (Vitaliano et al., 1998), reduced sleep (Shakhar et al., 2007), and type 2 diabetes (Shakhar et al., 2007), suggesting that many within even the general population are compromised in their ability to eliminate CTC’s, and are therefore at increased risk of tumor development.

*HYPOTHESIS*

Past research has focused on reintroduction/reinfusion of the patient-derived primary site tumor cells shed during surgery via autologous blood transfusions (Waters et al., 2012; Klein, 1995; Raghavan and Marik, 2005). That is, non-donor-derived primary site tumor cells which are ‘salvaged’ from the operating site and are ‘salvage’ and are reinfused into the patient, potentially facilitating metastasis of the original malignancy. 

**Figure 1:**
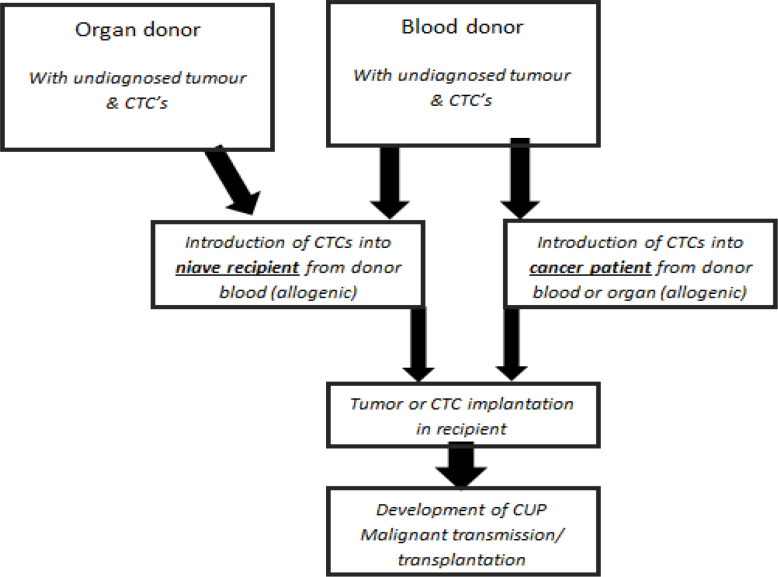
Haematogenic Cancer Transmission Scenarios

It is (thus) hypothesised, that the apparent contradictory results for occurrence of malignancies associated with peri-operative blood transfusions (Salunkhe et al., 2015; Vamvakas and Blajchman, 2009; Klein, 1995; Li et al., 2015; Waters et al., 2012; Vamvakas, 1995; Gakhar et al., 2013; Heiss et al., 1994), or occurrence of malignancies of unknown primary origin, may be elucidated further by proposing two additional distinct scenarios of haematogenic tumor transmission (Figure 1), namely:

1- Introduction of donor-derived CTCs from allogenic blood transfusions into known cancer surgery patients. That is, the donor has not yet presented with a sign or symptom of (i) recurrence (5yrs) (clinically dormant cancer) or (ii) first time cancer (pre-clinical cancer). Thus this may account for the presence/occurrence of a new malignancy/cancer of unknown primary origin (CUP) in the recipient.

2- Introduction of donor-derived CTCs during allogenic blood transfusions into niave/non-cancer patients. Neither individual (donor or recipient) having yet presented with signs or symptoms of cancer (clinical disease). Thus this may account for the occurrence of a new malignancy/cancer of unknown primary origin (CUP) in the recipient. 

Therefore, person-to-person transmission of cancer may arise via organ donation transmission of CTCs entrained in the organ vasculature or via allogenic blood donation transmission of CTCs directly at the time of transplantation surgery, together or independently.

*CONCLUDING REMARKS*

The evidence strongly supports tumor transmission from the allogenic organ donor during transplantation. It has been assumed that the recipient malignancy described in the literature was derived (solely) from the donor organ. However, we postulate that the donor-derived malignancies may have actually been derived from transmission of CTCs present in the blood that was either entrained in the donor organ vasculature or provided as an allogenic blood transfusion during the transplantation surgery.

While cancer transmission through tissue tumor cells is well accepted, to date, no study has yet explored the role of donor-derived CTCs in facilitating cancer (CUP) genesis in the recipient following either an organ donation or an allogenic blood donor transfusion or both.

The capacity of donated CTCs to proliferate in the recipient’s blood may not be dissimilar to donor leukocyte proliferative activity in GVHD, especially in those with reduced immune function (e.g. reduced NK cell activity). 

The risks of malignancy/cancer transmission from donors with clinically dormant cancers and pre-clinical cancers during organ donation or allogenic blood transfusions require urgent and immediate reassurance initiatives/measures. This article, in providing a perspective, focuses primarily on high-income nations and in turn heralding the even greater need for world-wide attention to the other members of our global family – the low-income nations. 

*Recommendations for consideration*

1. Donor clinical research to include (i) retrospective secondary analysis of 5-10 years blood transfusion data to assess relative cancer risk in recipients and (ii) prospective studies to create a robust and comprehensive set of metadata detailing donor and recipient details and relative cancer risk to improve our understanding and enable stringent hypothesis testing.

2. Donor exclusion criteria to be expanded to exclude any person with a history of cancer, whether or not they have been cancer free for 5 years (clinically dormant cancer patients). 

3. Donor exclusion criteria to be expanded to exclude any person who falls into the ‘at risk’ group for pre-clinical cancer, that is, above the age of 45, who begin to cross over into the various age-relate national (USA) cancer screening thresholds. 

4. Donor allogenic blood screening platforms be explored that include the capture, confirmation and categorisation of CTCs (pre-clinical cancer patients) that may complement existing allogenic blood transfusion protocols. 

It is noted that most current methodologies for detecting CTC’s are labor intensive and expensive, limiting the use of this important marker in lower income countries. However, technology in this field is rapidly expanding (Hendricks et al., 2020) and cost effective options are anticipated to be available in the not too distant future. 

5. Review blood leuko-reduction and irradiation protocols.

In context of the lengthy latency period of most human cancers before diagnosis, and the preponderance of ‘advanced cancer’ diagnoses, the likelihood of CTCs presenting in blood during this window period represents a unique opportunity to identify pre-clinical cancer in its early stages. Aside from also protecting organ and blood recipients from the risk of cancer transmission from clinically dormant cancer(s), with the emergence of CTC screening platforms on the horizon, these additional safety measure(s) will shift the focus to early detection and management of cancer in the general population.

## Author Contribution Statement

Conceptualisation; EM, Writing original draft manuscript; EM. Data/reference curation EM,RG. Administration RG. Preparation of revised manuscript; RG, Review & submission of final draft of manuscript, EM, RG.

## Acknowledgments

### Ethical statement

This article does not contain any studies involving human (or animal) participants performed by any of the authors

### Conflict of interest

The authors have no conflict of interest.
